# Early experience with permanent pacemaker implantation at a tertiary hospital in Nigeria

**DOI:** 10.11604/pamj.2020.36.177.24425

**Published:** 2020-07-13

**Authors:** Uvie Ufuoma Onakpoya, Olugbenga Olalekan Ojo, Oghenevware Joel Eyekpegha, Abayomi Emmanuel Oguns, Anthony Olubunmi Akintomide

**Affiliations:** 1 Department of Surgery, College of Health Sciences, Obafemi Awolowo University, Obafemi Awolowo University Teaching Hospital, Ile-ife, Nigeria,; 2 Department of Surgery, Obafemi Awolowo University Teaching Hospital, Ile-ife Nigeria,; 3 Department of Medicine, College of Health Sciences, Obafemi Awolowo University, Obafemi Awolowo University Teaching Hospital, Ile-ife, Nigeria

**Keywords:** Pacemaker, bradyarrhythmia, complete heart block, single chamber (VVIR), dual chamber (DDDR)

## Abstract

**Introduction:**

artificial pacemakers generate electrical impulses and regulate the heart&acutes conduction system. They are often used to treat individuals with bradycardia. Permanent pacemaker implantation is a lifesaving procedure especially in patients with symptomatic bradyarrhythmias. The objectives was to evaluate the clinical attributes and outcomes of permanent pacemaker implantation in Ile-ife, Nigeria.

**Methods:**

we retrospectively reviewed medical records of 22 patients who had pacemaker implantation from January 2015 to December 2019. Patient´s demographics, clinical presentation, diagnosis, comorbidities, type of device, complications and long-term follow up were studied.

**Results:**

sixteen males (72.7%) and 6 females (27.3%) were recruited into the study with ages ranging between 54 and 84 years and a mean of 70.3 +8.7 years. The commonest symptom was easy fatigability (45.5%) followed by syncope (31.8%). The main indication for permanent pacemaker implantation was complete heart block (86.4%). Seventeen (77.3%) patients had hypertension as the comorbidity present at diagnosis. Single chamber (VVIR) pacemaker was implanted in 13(59.1%) patients while dual chamber (DDDR) was implanted in 9(40.9%) patients. Hematoma, pneumothorax and acute lead dislodgement were the complications observed in 3 patients. There was no statistical significance between the type of device implanted and the occurrence of complications, p-value 0. 186. There was no mortality and 15 patients (68.2%) are currently attending regular 6 monthly follow-up.

**Conclusion:**

complete heart block is the most common indication for permanent pacemaker implantation and the procedure is safe with minimal complications and satisfactory outcomes.

## Introduction

Permanent pacemaker implantation (PPI) is a lifesaving procedure especially in patients with bradyarrhythmias. Bradyarrhythmias are most commonly caused by failure of impulse formation (sinus node dysfunction) or by failure of impulse conduction over the atrioventricular (AV) node/His-Purkinje system [1]. Amongst the various AV conduction abnormalities, complete third-degree AV block (AVB) with or without symptoms has been demonstrated to be the most common indication for pacemaker implantation in Nigeria and West Africa at large [2-4]. Sinus node dysfunction otherwise known as Sick sinus syndrome is the predominant indication for cardiac pacing in Western population [5]. Other indications for permanent pacemaker implantation include; symptomatic second degree AV bock (Mobitz type I and II), chronic bi-fascicular block, persistent second degree AV block post myocardial infarction, neurocardiogenic syncope etc. In the paediatric age group, congenital complete heart block and more often persistent heart block post cardiac surgical interventions are prominent indications for permanent pacemaker implantation. Since the introduction of transvenous endocardial pacing in 1958, the field of cardiac pacing has benefited greatly from advances made in electronics, computer technology, power sources, and miniaturization [1]. There has been an increasing number of indications for cardiac device implantation including permanent pacemakers over the years [6, 7] and this has become evident in our environment too [3, 8]. Most permanent pacemakers are implanted tranvenously under local anaesthesia with or without sedation via the cephalic, axillary or subclavian veins. The cephalic access is achieved via a cut down technique and this route avoids the risk of pneumothorax which can occur with the subclavian puncture technique. This technique however requires a patent cephalic vein, which is not always the case in older patients and theoretically the relative extensive dissection when compared to a medial subclavian puncture might lead to a higher incidence of pacemaker pocket hematoma. The axillary vein route for transvenous pacemaker implantation avoids the narrow costoclavicular angle which is a potential spot for pacemaker lead compression/fracture when employing the subclavian puncture technique. Regardless of the access vein for transvenous endocardial pacing, notable procedural and immediate post procedure complications of permanent pacemaker implantation include; pneumothorax, hemothorax, air embolism, cardiac perforation, pacemaker pocket hematoma, pocket site infection, acute lead dislodgement, diaphragmatic pacing and sepsis. The aim of our study is to evaluate the clinical attributes and early outcomes of permanent pacemaker implantation in a tertiary hospital in Nigeria.

## Methods

This was a retrospective study of patients who underwent primary transvenous permanent pacemaker implantation for various indications between January 2015 and December 2019 at our federally funded Teaching Hospital. All the patients in this study had transvenous permanent pacemaker implantation under local anesthesia. The Seldinger puncture technique was used to access the subclavian vein and the left subclavian vein was our first choice. Patients requiring secondary implantation and change of pacemaker pulse generator were exempted from this study. Patient´s demographics, clinical presentation, diagnosis, comorbidities, type of device, complications, and long-term follow up were studied. Data was obtained from the hospital medical records and analyzed with the SPSS statistical package (version 16.0). The student t test was used to compare continuous variables and the χ^2^or Fischer´s exact test for categorical variables. A p value of < 0.05 was considered statistically significant.

## Results

Twenty-two patients had permanent pacemaker implantation (PPI) during the 5-year review period. There were 16 males (72.7%) and 6 females (27.3%) in the study. The age range was between 54-84 years with a mean of 70.3 +8.7. The most common symptom at presentation was easy fatigability occurring in 10(45.5%) patients followed by syncope 7(31.8%) (Table 1). Complete heart block was the most common indication for permanent pacemaker implantation, and this was observed in 19(86.4%) patients. Most patients had hypertension 17(77.3%) as the comorbidity present at implantation, 2(9.1%) patients had both hypertension and diabetes mellitus. Single chamber (VVIR) pacemaker was implanted in 13(59.1%) patients while dual chamber (DDDR) was implanted in 9(40.9%) patients. Pacemaker pocket hematoma, pneumothorax and acute lead dislodgement were the complications observed in 3 patients (Table 1). There was no statistical significance between the type of device implanted and the occurrence of complications, p-value 0.186 (Fisher´s exact) Table 2. There was no in-hospital or 30 day mortality, all patients attended follow-up clinics up to 6 weeks post procedure, 6 patients have been transferred out to the referring hospital for continuation of post device care and 1 patient was lost to follow up. Fifteen patients (68.2%) are currently attending regular 6 monthly follow-up clinics at our hospital.

**Table 1:** patient characteristics, indication and complications

**Sex**		Frequency	%
	Male	16	72.7
	Female	6	27.3
**Age**	Mean age 70.3 ± 8.7 years (Range= 54-84years)		
**Presenting complaint**	Asymptomatic	3	13.6
	Easy Fatigue	10	45.5
	Dizziness	1	4.5
	Syncope	7	31.8
	Seizure	1	4.5
**Co-morbidities**	Hypertension	17	77.3
	Diabetes	2	9.1
	CVD (stroke)	1	4.5
	?? None	2	9.1
**Indication**	Complete heart block	19	86.4
	Sick sinus syndrome	1	4.5
	2°AVB (Mobitz type 2)	2	9.1
**Complications**		**3/22**	**13.6**
	Acute lead dislodgement	1	4.5
	Pocket hematoma	1	4.5
	Pneumothorax	1	4.5

CVD = cerebrovascular vascular disease, AVB = atrioventricular block

**Table 2:** complications based on type of device

Device	Complication		Total(n)	P-value
	yes	no		
VVIR	3	10	13	*0.186
DDDR	0	9	9	

* P value 0.186(Fischer exact test) VVIR = Single chamber ventricular pacing mode, DDDR= Dual chamber pacing mode

## Discussion

The male preponderance in our patients is in keeping with reports in other studies [5, 9, 10] while the mean age of patients in this study was 70.3 +8.7 with most patients between the ages of 70-79 years (40.9%). This is consistent with findings in other studies and also shows the increasing need of pacemaker implantation in older patients [3,11, 12]. The most common symptom at presentation was easy fatigability occurring in 10 (45.5%) patients followed by syncope in seven (31.8%) patients which is at variance with findings in other studies [11, 13]. In the study by Aktoz *et al*. [11] syncope was the main symptom in patients requiring permanent pacemaker implantation. Falase *et al*. [13] also found syncope (49%) as the most common clinical indication for permanent pacemaker insertion. This difference might be due to the fact that our study population is small. It remains to be seen if this trend will change in our subsequent studies with larger patient numbers towards what obtains in most other studies. Complete heart block was the most common indication for permanent pacemaker implantation. This has also been demonstrated in a vast majority of studies [2, 3, 10, 11, 14]. Hypertension was the most frequent co-morbidity in this study, which is consistent with previous findings in our environment [8, 10]. One patient suffered a stroke while waiting to accept the need for a permanent pacemaker implantation. Thomas *et al*. [8] had earlier admonished attending physicians of the need to balance medications to ensure an adequate and sustained blood flow to the vital organs like the brain and kidneys. The stroke in this patient could have been due to aggressive pharmacological lowering of the patient´s blood pressure. Single chamber (VVIR) pacemaker was implanted in almost two-thirds of patients while the rest had dual chamber implantation. The higher number VVIR mode in our study is in keeping with the findings of Aktoz *et al*. [11] and most reports from West Africa [2, 3, 15]. The choice of pacemaker mode/type in our patients was initially influenced by the cost because; the dual chamber device was far more expensive than the single chamber device until recent times when the cost difference was reduced by the manufacturers. This has led to a recent steady increase in the number of dual chamber pacemaker implantation in our centre and this is fast becoming the pacing mode of choice in some centres in West Africa [13] as it is in most developed countries [5, 14]. Complications observed in this series were acute lead dislodgement, pacemaker pocket hematoma and pneumothorax in one patient each. Lead repositioning was successfully done in the patient with lead displacement. The pacemaker pocket hematoma and pneumothorax were evacuated with satisfactory outcomes. All three complications occurred in patients who had single chamber pacemaker implantation however this was not statistically significant (p-value 0.186). This supports Aggarwal´s [16], finding of no difference in early complication rate between dual and single chamber systems. There was no procedure related mortality and most of the patients are being followed up in the pacemaker clinic.

## Conclusion

Elderly hypertensive patients with complete heart block are the most common indication for permanent pacemaker implantation in our environment. The implantation procedure is safe (regardless of the device type) with minimal complications and satisfactory outcomes.

### What is known about this topic

- Complete heart block is the commonest indication for permanent pacemaker insertion (PPI) in the West African sub-region;
- Syncope is the most frequent clinical indication for PPI.

### What this study adds

- Easy fatiguability is fast becoming a prominent presenting symptom in patients who eventually require PPI;
- There is a steady increase in the number of dual chamber pacemaker implantation in West Africa.
